# Identification of Epidermal Growth Factor Receptor Tyrosine-Kinase Mutations in Non-small Cell Lung Cancer: Testing Platform Matters

**DOI:** 10.7759/cureus.7316

**Published:** 2020-03-18

**Authors:** Shipra Gandhi, Ankita Kapoor, Grace Dy

**Affiliations:** 1 Oncology, Roswell Park Cancer Institute, Buffalo, USA; 2 Internal Medicine, Rochester General Hospital, Rochester, USA; 3 Thoracic Oncology, Roswell Park Cancer Institute, Buffalo, USA

**Keywords:** epidermal growth factor receptor, non-small cell lung cancer, tyrosine kinase inhibitor, next generation sequencing, erlotinib

## Abstract

Personalized medicine using targeted therapies has revolutionized the management of non-small cell lung cancer (NSCLC) in the past decade. The discovery that sensitizing epidermal growth factor receptor (EGFR) mutations are predictive for therapeutic benefit from EGFR tyrosine kinase inhibitors (TKIs) such as erlotinib marked the beginning of a new era in lung cancer therapeutics. Indeed, EGFR mutation testing is category A recommendation at the time of diagnosis for patients presenting with advanced-stage NSCLC.

In our case, the original report of EGFR mutation testing using pyro-sequencing from the initial biopsy was reported out as wild-type/no mutation seen in the hot spots. However, the tumor had a long duration of response to erlotinib but later developed resistance, hence there was a high index of suspicion. Consequently, it was decided to retest the tumor with more sensitive technology. Next generation sequencing identified exon 19 deletion - a sensitizing mutation. This explained the excellent response on initiating erlotinib, however, exon 21 mutation was also reported which confers resistance to TKI. The case shows that test sensitivity can have a great impact on treatment decisions and if there is a high index of suspicion, initial testing and, or retesting using newer more sensitive technology should be considered.

## Introduction

Personalized medicine using targeted therapies has revolutionized the management of non-small cell lung cancer (NSCLC) in the past decade. Better understanding of the molecular pathways and specific genetic alterations involved in carcinogenesis has led to significant advances in the development of targeted therapies. The discovery that sensitizing epidermal growth factor receptor (EGFR) mutations are predictive for therapeutic benefit from EGFR tyrosine kinase inhibitors (TKIs) such as erlotinib marked the beginning of a new era in lung cancer therapeutics [1,2]. Indeed, EGFR mutation testing is category A recommendation at the time of diagnosis for patients presenting with advanced-stage NSCLC [3].

Various methods can be used to ascertain EGFR mutation status in the tumor. We describe a patient deemed to have wild-type EGFR by hotspot pyro-sequencing analysis. Retesting using next-generation sequencing revealed the presence of TKI-sensitive exon 19 deletion. This case illustrates the significance of retesting if there is a high index of suspicion and highlights challenges in clinical management that medical oncologists should be aware of.

## Case presentation

**Figure 1:**
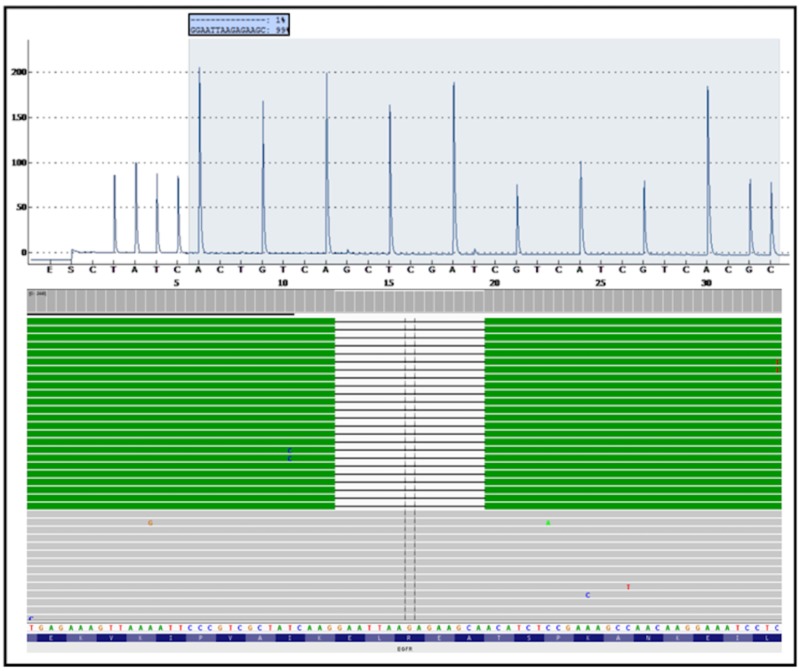
EGFR exon 19 pyro-gram and Miseq BAM pileup (NGS format of aligned sequences) in the re-biopsy specimen. There are no apparent aberrancies in the pyro-gram shown in the upper half of the figure. Within the BAM pileup in the lower half of the figure, the white spaces between the green rows represent a 15-base deletion. The deletion is detected at 5% allele frequency. The solid grey rows represent sequence that does not contain a deletion. EGFR: Epidermal growth factor receptor; NGS: Next-generation sequencing.

**Figure 2:**
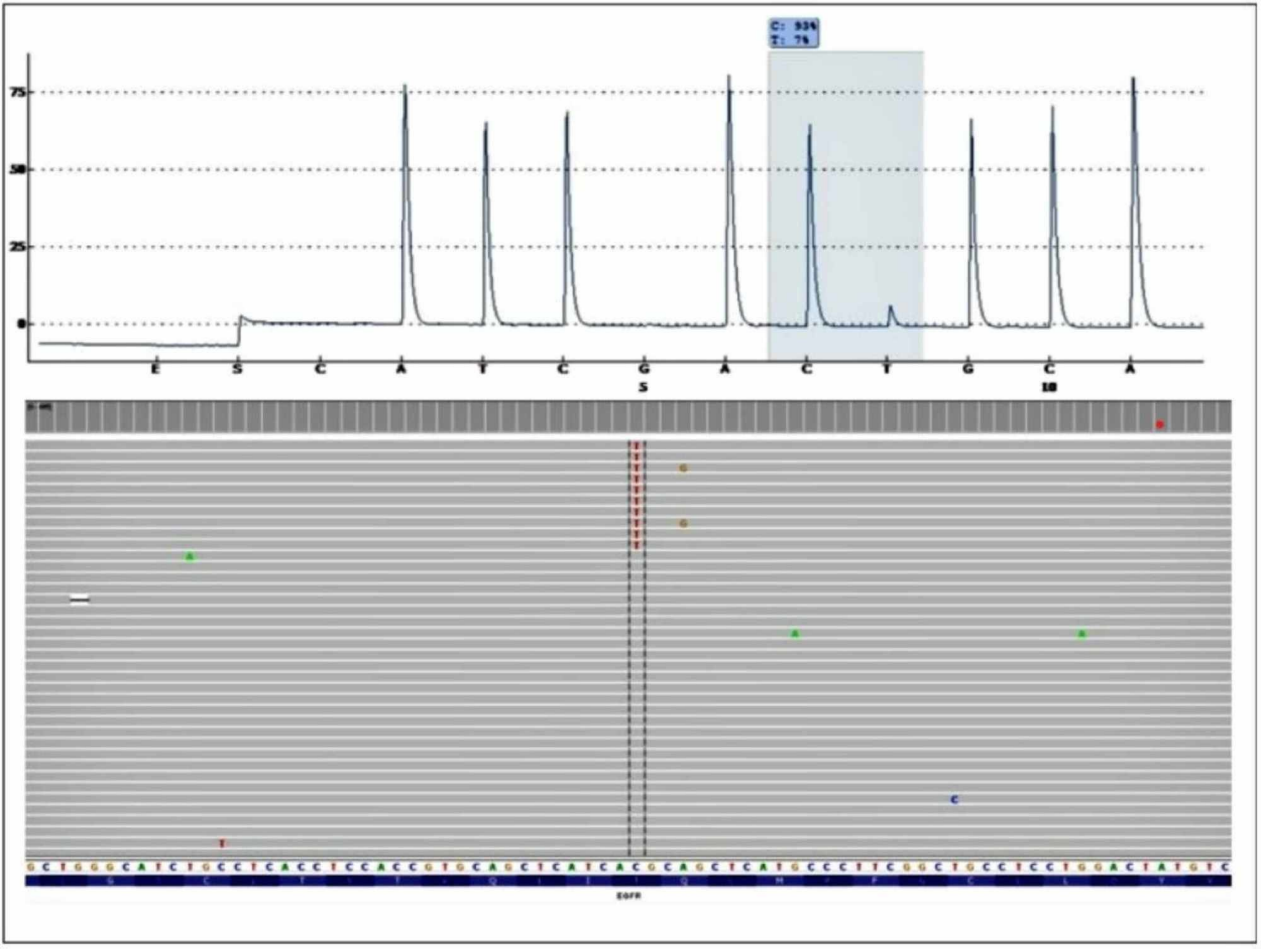
EGFR exon 790 pyro-gram and Miseq BAM pileup (NGS format of aligned sequences) in the re-biopsy specimen. An equivocal aberrancy is noted at the 790 position of the pyro-gram at a 7% frequency, which is below the 10% limit of detection of this laboratory developed test. A single base substitution indicative of a T790M mutation is identified in the MiSeq BAM pileup at 4% allele frequency. EGFR: Epidermal growth factor receptor; NGS: Next-generation sequencing.

We present a case of a 65-year-old nonsmoker, African-American lady who presented with cough and shortness of breath in 2009. CT chest and thoracentesis confirmed left-sided malignant pleural effusion. Cytology confirmed lung adenocarcinoma, no mutations were detected by a validated hot-spot pyro-sequencing test in the specimen. Neither ALK nor ROS1 translocations were present. She received platinum and pemetrexed followed by pemetrexed maintenance therapy for nearly three years which was eventually discontinued due to adverse effects. Erlotinib 150 mg was then started. There was significant tumor response upon follow-up imaging studies on erlotinib. Her disease remained stable while on therapy for nearly 18 months until disease progression was demonstrated on the CT scan. Repeat biopsy was obtained for molecular profiling for analyzing mutations using next-generation sequencing technologies. This revealed the classical exon 19 deletion in EGFR as well as the acquired T790M resistance mutation, at allele frequencies of 5% and 4%, respectively as shown in Figure 1 and Figure 2. Indeed, pyro-sequencing performed again on this latest specimen also did not detect the mutation. She was subsequently treated with four doses of nivolumab, which was stopped due to progression. She was subsequently again started on erlotinib for couple of months and then died due to progressive disease and poor performance status.

## Discussion

Epidermal growth factor receptor (EGFR), a 1186 amino acid trans-membrane receptor protein consists of an extracellular region with a ligand-binding domain and an intracellular region with tyrosine kinase and regulatory domains. Several ligands bind to the receptor, instigating homologous and/or heterologous dimerization and subsequent auto-phosphorylation of the intracellular domain, leading to a cascade of signal transduction resulting in cell proliferation and survival, inhibiting oncogenesis and hence, activating angiogenesis [4, 5]. The development of molecularly targeted therapeutic agents has revolutionized the outcome of advanced NSCLC. Afatinib, gefitinib, and erlotinib were the tyrosine kinase inhibitors (TKIs) that have been approved for the treatment of advanced NSCLC back in 2013 based on standard of care in the second-line setting after progression on chemotherapy in the metastatic setting [6, 7]. This patient was started on erlotinib in 2013 appropriately, however, since then, the first-line management of advanced NSCLC, EGFR-mutation positive has changed. Based on improvement in progression free and overall survival (38.6 versus 31.8 months) in EGFR-mutation positive advanced NSCLC compared to other EGFR inhibitors, osimertinib is now the standard first-line treatment in this setting [8-10].

The need for EGFR mutation testing in advanced NSCLC prior to second-line or subsequent cycles of chemotherapy remains unclear [11-13]. In the second-line, third-line, or maintenance settings, it appears that treatment with a TKI benefits all patients in terms of progression-free survival (PFS) and overall survival (OS) regardless of EGFR mutation status or patient characteristics. Plausible explanations include a change in mutational status after platinum-based chemotherapy regimens or another pathway superseding to make the patient’s tumor more sensitive to an EGFR inhibitor, thus producing a benefit in response, and survival. The IPASS (Iressa Pan-Asia Study) trial provides strong evidence for testing for EGFR in the first-line setting [14]. The INTEREST (Iressa Non-Small Cell Lung Cancer Trial Evaluating Response and Survival Against Taxoere) trial, a randomized phase III trial showed that there was no difference in response among patients with stage IV adenocarcinoma treated with gefitinib versus docetaxel in the second-line setting [15].

Therapy with EGFR TKIs might not be curative in long run in many cases even with initial excellent response with sensitizing EGFR mutations in NSCLC patients as they tend to develop resistance which leads to disease progression. The resistance mechanisms identified are secondary EGFR mutations, most commonly point mutation in T790M on exon 20, MET oncogene amplification or rarely histological small cell lung cancer transformation [10, 16, 17].

Various methodologies with different sensitivities have been devised to detect EGFR mutations. Next-generation sequencing (NGS) methodology, massively parallel sequencing, was validated in a study by Querings et al., which reported a 100% success rate of this method to detect low-frequency EGFR mutations compared with 89% for pyro-sequencing, a non-electrophoretic sequencing technology employing luminometric detection and 67% for direct sequencing [18].

As in our case, the original report of EGFR mutation test using pyro-sequencing from the initial biopsy was reported out as wild-type/no mutation seen in the hot spots. However, the tumor had a long duration of response to erlotinib but later developed resistance, hence there was a high index of suspicion. Consequently, it was decided to retest the tumor with more sensitive technology. Next generation sequencing identified exon 19 deletion, a sensitizing mutation. This explained the excellent response on initiating erlotinib, however, exon 21 mutation was also reported which confers resistance to TKI. The case shows that test sensitivity can have a great impact on treatment decisions and if there is a high index of suspicion, initial testing and, or retesting using newer more sensitive technology should be considered. This is especially important in the current era where osimertinib has been approved in the first-line setting with a PFS of 18.9 months [8].

## Conclusions

Our case illustrates the importance of knowing the laboratory method used in establishing the molecular profile of each patient. When there is a high-index of suspicion, retesting using more novel techniques, such as next generation sequencing, should be considered as the results can significantly impact the therapeutic choices available to a patient.
